# Optimal Timing of Invasive Coronary Angiography following NSTEMI

**DOI:** 10.1155/2020/8513257

**Published:** 2020-03-03

**Authors:** Thabo Mahendiran, David Nanchen, David Meier, Baris Gencer, Roland Klingenberg, Lorenz Räber, David Carballo, Christian M. Matter, Thomas F. Lüscher, Stephan Windecker, François Mach, Nicolas Rodondi, Olivier Muller, Stephane Fournier

**Affiliations:** ^1^Department of Cardiology, Lausanne University Center Hospital, Lausanne, Switzerland; ^2^Center for Primary Care and Public Health (Unisanté), University of Lausanne, Lausanne, Switzerland; ^3^Department of Cardiology, Geneva University Hospitals, Geneva, Switzerland; ^4^Department of Cardiology, Kerckhoff Klinik, Bad Nauheim, Germany; ^5^Department of Cardiology, University Hospital of Zurich, Zurich, Switzerland; ^6^Center for Molecular Cardiology, University of Zurich, Zurich, Switzerland; ^7^Imperial College and Royal Brompton & Harefield Hospitals, London, UK; ^8^Department of Cardiology, Inselspital, Bern University Hospital, University of Bern, Bern, Switzerland; ^9^Department of General Internal Medicine, Inselspital, Bern University Hospital, University of Bern, Bern, Switzerland; ^10^Institute of Primary Health Care (BIHAM), University of Bern, Bern, Switzerland; ^11^Division of Cardiology, Department of Advanced Biomedical Sciences, Federico II University, Via S. Pansini, 5, 80131 Naples, Italy

## Abstract

**Objective:**

To obtain a real-world perspective of the optimal timing of angiography performed within 24 hours of admission with non-ST elevation myocardial infarction (NSTEMI).

**Background:**

Current guidelines recommend angiography within 24 hours of hospitalisation with NSTEMI. The recent VERDICT trial found that angiography within 12 hours of admission with NSTEMI was associated with improved cardiovascular outcomes among high-risk patients. We compared the outcomes of real-world NSTEMI patients undergoing angiography within 12 hours of admission with those of patients undergoing angiography 12 to 24 hours after admission.

**Methods:**

NSTEMI patients without life-threatening features who received angiography within 24 hours of admission were obtained from the SPUM-ACS registry, a cohort of consecutive patients admitted with acute coronary syndromes to four university hospitals in Switzerland. Cox models assessed for an association between door-to-catheter time and one-year major adverse cardiovascular events (MACE: cardiovascular mortality, myocardial infarction, and stroke).

**Results:**

Of 2672 NSTEMI patients, 1832 met the inclusion criteria. Among them, 1464 patients underwent angiography within 12 hours (12 h group) compared with 368 patients between 12 and 24 hours (12–24 h group). Multiple logistic regression identified out-of-hours admission as the only factor associated with delayed angiography. After 2 : 1 propensity score matching, 736 patients from the 12 h group and 368 patients from the 12–24 h group demonstrated no significant difference in rates of one-year MACE (7.7% vs. 7.3%, HR: 1.050, 95% CI 0.637–1.733, *p*=0.847). Stratification by GRACE score (>140 vs. ≤140) found no significant reduction in MACE among high-risk patients in the 12 h group (*p*=0.847). Stratification by GRACE score (>140 vs. ≤140) found no significant reduction in MACE among high-risk patients in the 12 h group (

**Conclusions:**

In an unselected real-world cohort of NSTEMI patients, angiography within 12 hours of admission was not associated with improved one-year cardiovascular outcomes when compared with angiography 12 and 24 hours after admission, even among high-risk patients.

## 1. Introduction

Current European and American guidelines recommend angiography within 24 hours of hospitalisation for patients with non-ST elevation myocardial infarction (NSTEMI). Key to these recommendations was the TIMACS trial (Timing of Intervention in Acute Coronary Syndromes) which found that invasive angiography within 24 hours of admission was associated with a reduced rate of recurrent ischemia at 6 months when compared with angiography ≥36 hours after admission [1]. Additionally, a reduced rate of the composite primary endpoint (death, myocardial infarction (MI), and stroke) was noted among patients with a GRACE score >140 receiving early angiography. Further support came from the RIDDLE-NSTEMI trial, which found that early angiography (median time 1.4 hours) following NSTEMI was associated with reduced mortality/recurrent MI at both 30 days and 1 year when compared with a delayed strategy (median time 61 hours) [2]. Meta-analyses have provided additional support for these recommendations [3,4]. Of note, Jobs et al. analysed data from eight studies (including TIMACS and RIDDLE-NSTEMI) and found reduced 6-month mortality among NSTEMI patients treated with an early invasive strategy [4].

The recent VERDICT (Very EaRly vs Deferred Invasive evaluation using Computerized Tomography) trial provided evidence of the potential benefits of even earlier intervention in high-risk patients. Patients without life-threatening features were randomised to receive angiography within 12 hours or between 48 and 72 hours. Among the early intervention group, patients with a GRACE score >140 were found to have a reduced rate of the primary composite endpoint (all-cause death, nonfatal recurrent MI, admission for refractory myocardial ischemia, or heart failure) at 4 years [5]. However, there are limitations in the design of the VERDICT trial that potentially reduce its applicability to clinical practice. Firstly, early angiography was delivered with a median door-to-catheter (DTC) time of 4.7 hours (IQR 3.0–12.2), compared to a delayed strategy with a median DTC time of 61.6 hours (IQR 39.4–87.8). This represents a substantial treatment delay that high-risk patients typically would not experience in routine clinical practice. Secondly, DTC times between 12 and 24 hours were not considered despite a significant proportion of patients in clinical practice likely experiencing a delay of this length.

The sheer volume of NSTEMI admissions makes the timely delivery of angiography a significant challenge for clinicians and the latest evidence from the VERDICT trial could be interpreted as supporting an even more challenging angiography target in high-risk NSTEMI.

We aimed to obtain a real-world perspective of the optimal timing of invasive angiography following NSTEMI among patients treated within the recommended 24-hour target. In a retrospective analysis of consecutive patients admitted with NSTEMI to four university hospitals across Switzerland, we compared one-year outcomes of patients receiving angiography within 12 hours with those of patients receiving angiography between 12 and 24 hours.

## 2. Materials and Methods

### 2.1. Patients

Data were obtained from the SPUM-ACS (Special Program University Medicine–Acute Coronary Syndromes) registry, a cohort of consecutive patients admitted with acute coronary syndromes (ACS) to four PCI-capable university hospitals in Switzerland. ACS was defined as the presence of symptoms consistent with angina pectoris and at least one of the following characteristics: ST-segment elevation or depression, T inversion or dynamic ECG changes, evidence of positive troponin, and/or known coronary heart disease (status after myocardial infarction, bypass surgery, or coronary angiography). All patients were aged 18 years or over with the only exclusion criteria being severe physical disability, inability to comprehend the study, or life expectancy of less than 1 year (for noncardiac reasons). Further details of this registry have been reported previously [6]. For the present study, patients hospitalised between 2009 and 2017 with a diagnosis of NSTEMI were selected. NSTEMI was defined as elevated troponin levels and the absence of ST elevation at the time of diagnosis. Among them, DTC time was calculated by subtracting the time of coronary catheter insertion from the time of hospitalisation. Patients without a DTC time, those receiving angiography greater than 24 hours after admission, and those presenting with very-high-risk criteria (cardiac arrest, systolic blood pressure < 90 mmHg, acute cardiac failure (Killip class III or IV), and ST elevation on ECG) were excluded.

### 2.2. Primary Endpoint

The primary endpoint was defined as one-year major adverse cardiovascular events (MACE), a composite of cardiovascular mortality, recurrent MI (using the universal definition of MI [7]), and stroke. The incidence of cardiovascular events during follow-up was ascertained by telephone consultation 30 days after discharge and in a clinical face-to-face visit at one year. When patients could not be reached for the one-year follow-up visit, medical information was obtained from primary care physicians, family members, hospital records, or a registry office. Three certified cardiologists adjudicated all cardiovascular events.

### 2.3. Statistical Analysis

Normally distributed, continuous variables are expressed as mean ± SD and compared using the 2-tailed Student *t*-test. Nonnormally distributed continuous variables are expressed as a median with interquartile range and analysed using the Mann–Whitney *U* test. Comparisons between categorical variables were performed using the Pearson *χ*2 test. Missing values in baseline clinical characteristics were treated with multiple imputation in order to create five imputed datasets. Baseline and treatment characteristics are presented for a single imputed dataset. Logistic and Cox regression analyses were performed on each imputed dataset before pooling of estimates as per Rubin's Rules [8]. Multiple logistic regression was used to identify independent factors associated with an angiography delay of 12–24 hours. These models included the following covariates: age, sex, hypertension, diabetes, previous cardiovascular disease, chronic lung disease, smoking status, heart rate, systolic blood pressure, ECG ischemia, anaemia, estimated glomerular filtration rate (eGFR), GRACE score, admission at night, and admission on weekend. A 2 : 1 propensity score-matched analysis with a nearest-neighbour matching algorithm was used to manage differences in baseline characteristics. Patients were matched for age, sex, diabetes, hypertension, hypercholesterolemia, previous MI, previous PCI, previous CABG, previous stroke, previous CVD, valvular disease, chronic lung disease, family history of CVD, systolic blood pressure, heart rate, BMI, anaemia, baseline eGFR, ECG ischemia, smoking, GRACE score, and LVEF. Cox proportional hazards models assessed for an association between DTC time and the clinical endpoints. Stratified Kaplan–Meier analysis was used to visualise intergroup differences for each clinical outcome over the follow-up period. A *p*-value <0.05 was defined as statistically significant. Statistical analysis was performed using R version 3.5.1.

## 3. Results

### 3.1. Patients

Of 2672 NSTEMI patients, 534 without a DTC time and 94 with a DTC time greater than 24 hours were excluded. A further 212 patients were excluded due to presence of very-high-risk criteria. Among the remaining 1832 eligible patients, 1464 patients received angiography within 12 hours of admission (12 h group) while 368 patients received angiography between 12 and 24 hours (12–24 h group). Missing values in baseline clinical characteristics were managed with multiple imputation (Supplementary [Supplementary-material supplementary-material-1]). Prior to matching, the groups differed significantly with regards to age, previous cardiovascular disease, and baseline eGFR. After 2 : 1 propensity score matching, 736 patients from the 12 h group and 368 patients from the 12–24 h group presented no significant differences in main baseline clinical characteristics (Table 1). The median follow-up time was 365.2 days (IQR 358.0–365.2) in both the 12 h and 12–24 h groups. The median DTC time was 3.5 hours (IQR 1.8–6.0) in the 12 h group and 16.8 hours (IQR 14.4–19.5) in the 12–24 h group. The mean GRACE score was 130.12 (SD 27.84) in the 12 h group and 129.21 (SD 29.07) in the 12–24 h group. In both groups, 36% of patients had a GRACE score over 140.

With regards to treatment, both groups in the matched population had over 99% compliance with dual antiplatelet therapy at the time of discharge, though a smaller proportion of patients in the 12–24 h group were prescribed prasugrel (Table 2). There was also a higher incidence of coronary thrombus in the early intervention group (21.1% vs. 13.2%,*p*=0.003). However, there were no other significant differences in treatment characteristics. Of note, there were no significant differences in the proportion of patients receiving stenting (12 h: 84.8% vs. 12–24 h: 82.3%), balloon angioplasty only (12 h: 5.0% vs. 12–24 h: 5.4%), or CABG following angiography (12 h: 1.9% vs. 12–24 h: 1.6%) (*p*=0.606). As reported previously, there was strong adherence to ACS prescription guidelines [6].

### 3.2. Factors Associated with Angiography Delays of 12–24 Hours

Within the 12–24 h group, a much higher proportion of admissions occurred on weekday nights (12 h: 18.2% vs. 12–24 h: 44.3%) and on weekends (12 h: 15.5% vs. 12–24 h: 25.3%) (Table 2). This finding was reflected in the timing of admissions, with patients in the 12 h group typically admitted in the morning (median time 10 : 21, IQR 07 : 55–13 : 52), compared with much later admissions in the 12–24 h group (median time 18 : 02, IQR 14 : 10–20 : 40). After adjustment for a range of baseline characteristics including age, past medical conditions, and GRACE score, logistic regression identified admission at night (OR 4.462, 95% CI 3.221–6.181, *p* < 0.001) or on weekends (OR 1.503, 95% CI 1.031–2.191, *p*=0.032) as the only significant factors associated with an angiography delay of 12–24 hours. On average, patients in the 12–24 h group remained in hospital for one day longer than those in the 12 h group (12 h: median 2.0 days, IQR 1.0–4.4 vs. 12–24 h: median 3.0 days, IQR 1.2–5.0, *p* < 0.001).

### 3.3. Outcomes in Overall Population

**Figure 1:**
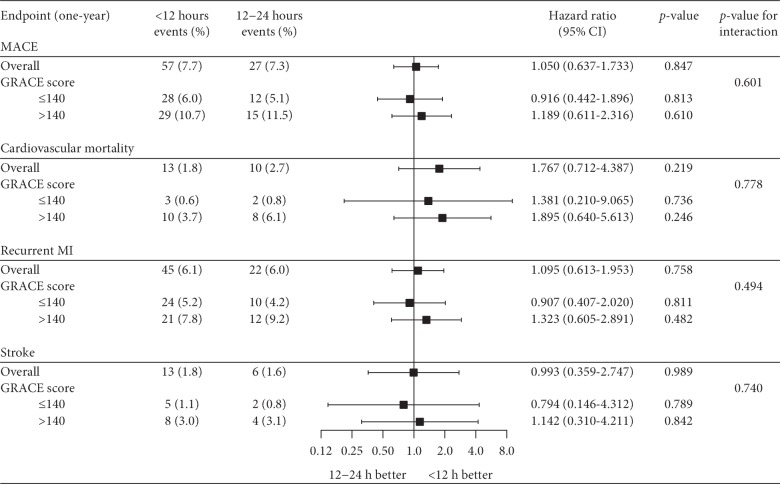
Forest plot of one-year endpoints in the overall matched population and after stratification by GRACE score. MACE represents a composite of cardiovascular mortality, recurrent MI, and stroke. For each one-year clinical endpoint, event rates are presented for the <12 h and 12–24 h groups along with the hazard ratio and *p*-value from the corresponding Cox proportional hazards model. For the GRACE score subgroup analysis, the *p*-value for interaction is presented. CI = confidence interval; GRACE = Global Registry of Acute Coronary Events; MACE = major adverse cardiovascular events; MI = myocardial infarction.

**Figure 2:**
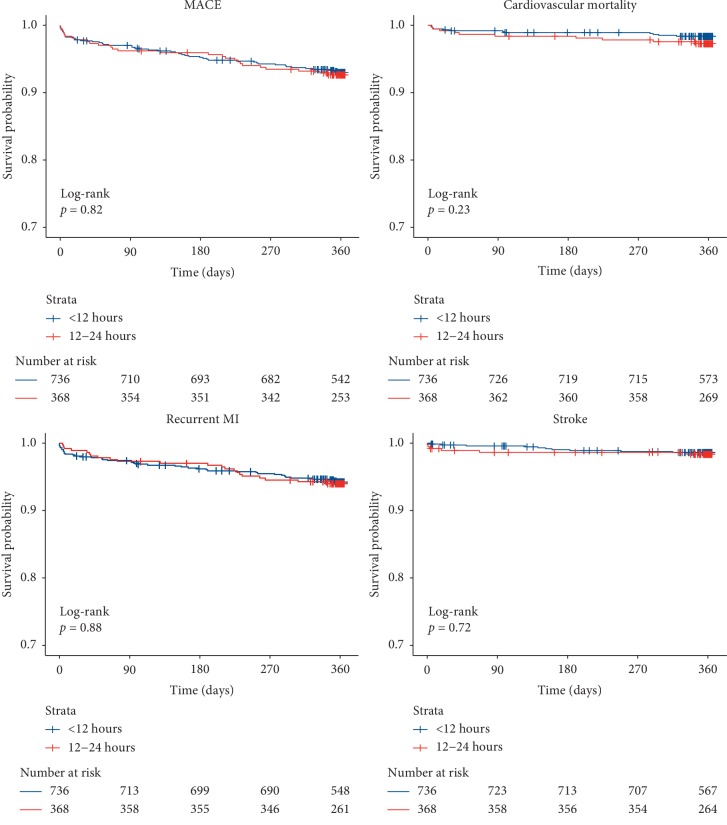
Kaplan–Meier plots stratified by door-to-catheter time for one-year endpoints. MACE represents a composite of cardiovascular mortality, recurrent MI, and stroke. For each endpoint, the *p*-value from the corresponding log-rank test is presented. MACE = major adverse cardiovascular events; MI = myocardial infarction.

At one year, the primary endpoint (MACE) occurred in 7.7% of patients in the 12 h group compared with 7.3% of patients in the 12–24 h group (HR: 1.050, 95% CI 0.637–1.733, *p*=0.847). There was increased cardiovascular mortality in the 12–24 h group; however, this was not statistically significant (1.8% vs. 2.7%, HR: 1.767, 95% CI 0.712–4.387, *p*=0.219). Additionally, there were no significant differences between groups with regards to recurrent MI (6.1% vs. 6.0%, HR: 1.095, 95% CI 0.613–1.953,*p*=0.758) or stroke (1.8% vs. 1.6%, HR: 0.993, 95% CI 0.359–2.747, *p*=0.989) (Figure 1). These findings were corroborated with Kaplan–Meier analyses which demonstrated no significant differences between groups with regards to MACE (*p*=0.82), cardiovascular mortality (*p*=0.23), recurrent MI (*p*=0.88), or stroke (*p*=0.72) at one year (Figure 2).

### 3.4. Outcomes in High-Risk Patients

**Figure 3:**
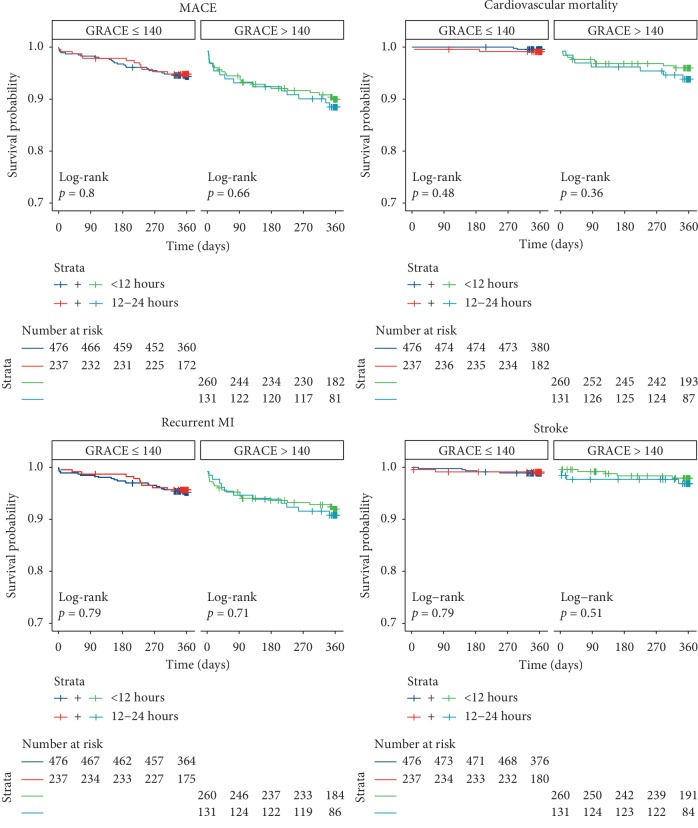
Kaplan–Meier plots stratified by door-to-catheter time and GRACE score for one-year endpoints. MACE represents a composite of cardiovascular mortality, recurrent MI, and stroke. For each endpoint, the *p*-value from the corresponding log-rank test is presented. GRACE = Global Registry of Acute Coronary Events; MACE = major adverse cardiovascular events; MI = myocardial infarction.

Among patients with a GRACE score >140 (12 h: 36.4% vs. 12–24 h: 36.0%), there was no significant difference between groups with regards to the rate of MACE (10.7% vs. 11.5%, HR: 1.189, 95% CI 0.611–2.316, *p* for interaction = 0.601). There was increased cardiovascular mortality (3.7% vs. 6.1%, HR: 1.895, 95% CI 0.640–5.613) in the 12–24 h group; however, this difference was not statistically significant (*p* for interaction = 0.778). The rates of recurrent MI (7.8% vs. 9.2%, HR: 1.323, 95% CI 0.605–2.891) and stroke (3.0% vs. 3.1%, HR: 1.142, 95% CI 0.310–4.211) were also not significantly different in the 12 h and 12–24 h groups at one year (*p* for interaction = 0.494 and 0.740, respectively) (Figure 1). This absence of benefit among patients with a GRACE score >140 was confirmed with KM analyses for all clinical endpoints (Figure 3).

## 4. Discussion

This study demonstrates that in a matched cohort of NSTEMI patients without life-threatening features, patients receiving invasive angiography within 12 hours had similar one-year cardiovascular outcomes to those treated between 12 and 24 hours. This finding was independent of GRACE score, with high-risk patients (GRACE score >140) having similar outcomes in both the 12 h and 12–24 h groups.

### 4.1. Relevance to Routine Clinical Practice

The VERDICT trial was key to the conception of this study. It demonstrated that angiography within 12 hours was only associated with improved long-term outcomes among non-ST elevation acute coronary syndrome (NSTE-ACS) patients with a GRACE score >140 [5]. However, direct comparison of our results with those of the VERDICT trial should be limited due to an important difference between the delayed intervention groups. In the VERDICT trial, early angiography was compared with delayed angiography conducted between 48 and 72 hours (median DTC time 61.6 hours, IQR 39.4–87.8). In routine clinical practice, high-risk NSTE-ACS patients typically do not experience such long delays, with current guidelines recommending angiography within 24 hours [9, 10]. Thus, this key finding from the VERDICT trial should not come as a surprise. However, our study addresses a gap in the VERDICT trial design by assessing the impact of delays experienced within the recommended 24-hour window. It is not uncommon for patients hospitalised with NSTEMI to experience delays prior to angiography [11, 12]. In this study, admission out-of-hours was the factor most strongly associated with delays of greater than 12 hours. Importantly, our data suggest that these delays do not have a significant association with one-year cardiovascular outcomes, provided that angiography is performed within the guideline-recommended 24-hour timeframe. Support for these findings come from the TIMACS trial which noted no significant difference in 6-month outcomes between patients treated within 6 hours, 6 to 12 hours or 12 to 24 hours [1]. Additionally, post-hoc analysis of the ACUITY trial (Acute Catheterization and Urgent Intervention Triage strategY) found no significant difference in 30-day or one-year outcomes between patients treated within 8 hours or between 8 and 24 hours [13].

It should be noted that the event rates in this study were lower than reported in previous studies, which may reflect an overall lower-risk NSTEMI population. For example, this study excluded patients presenting with any life-threatening features (as per European/American guidelines), whereas trials such as TIMACS and ACUITY included many of these patients [1, 13]. Additionally, compared with the present study, the VERDICT trial included patients with a higher-mean GRACE score (141 vs. 130) and a higher percentage of GRACE scores >140 (49% vs. 36%), suggesting an overall higher-risk population [5]. The presence of a lower-risk NSTEMI population in the present study may help explain why the referral rate for CABG was lower than reported in other studies.

### 4.2. Benefits of Early versus Delayed Angiography

The theoretical benefit of early angiography is the early identification of significant lesions facilitating early revascularisation and salvage of ischemic myocardium. Additionally, early angiography can promote early discharge. In this study, patients in the 12–24 h group had significantly longer hospital stays likely reflecting the delay in receiving angiography. On the other hand, delayed angiography following NSTEMI may provide adequate time for optimal medical treatment in order to decrease thrombus burden, improve plaque stability, and reduce subsequent stent thrombosis risk. The presence of coronary thrombus at the time of angiography is known to worsen outcomes following ACS [14]. Furthermore, in vitro models have shown that P2Y_12_ agents can disrupt and even reverse thrombus stability [15, 16]. Longer pretreatment with P2Y12 agents is also associated with improved coronary perfusion before PCI [17]. In this study, the proportion of patients with coronary thrombus was significantly higher in the early intervention group. Thus, it could be hypothesised that angiography deferred to between 12 and 24 hours may have provided time for the beneficial effects of medical therapy to be seen.

### 4.3. Limitations

This study is limited by its retrospective and observational design. As it is not possible to exclude the possibility of selection bias, these results should be considered as hypothesis generating. However, the SPUM-ACS registry represents a multicentre cohort of consecutive patients hospitalised with ACS. Additionally, given the very few exclusion criteria, we feel that the risk of bias is limited and that patients included in this study are likely representative of those encountered in routine clinical practice. Another limitation was the modestly-sized, imbalanced groups. To counter this, we applied 2 : 1 propensity score matching to create statistically balanced groups for comparison. However, it is possible that the small sample sizes in this study may have reduced the chance of detecting significant intergroup differences, particularly in the stratified GRACE score analysis. There was a higher rate of one-year cardiovascular mortality among patients receiving angiography between 12 and 24 hours, albeit nonsignificant. Interpretation of this component of the composite endpoint should be performed with caution given the small sample size. A further limitation is that the definition of MI changed during the study period (2009–2017), and thus a degree of heterogeneity could be present in the population. There were also relatively low prescription rates of ticagrelor and prasugrel in this study. This was likely reflective of the long study period which encompassed a shift in practice from higher rates of clopidogrel use at the start of the study period towards higher rates of more potent P2Y_12_ agents by the end of the study. A further limitation was the absence of detailed information on coronary thrombosis. A higher rate of coronary thrombosis was detected among patients in the 12 h; however, no information was available on thrombosis of preexisting coronary stents. Finally, with a median follow-up period of 365 days, this study cannot exclude the possibility of differences in outcomes beyond this timepoint.

## 5. Conclusion

In this real-world cohort of NSTEMI patients, angiography within 12 hours of hospitalisation was not associated with improved one-year cardiovascular outcomes when compared with angiography between 12 and 24 hours, even among patients with an elevated GRACE score.

## Figures and Tables

**Table 1:** Baseline characteristics of unmatched and matched populations. Propensity score 2 : 1 matching performed using a nearest neighbour matching algorithm.

Baseline characteristics	Unmatched			Matched		
	<12 h (*n* = 1464)	12–24 h (*n* = 368)	*p*-value	<12 h (*n* = 736)	12–24 h (*n* = 368)	*p*-value
Age, median (IQR)	63.05 (54.10, 72.40)	64.65 (55.88, 75.10)	**0.015**	65.00 (56.00, 75.00)	64.65 (55.88, 75.10)	0.853
Female (%)	308 (21.0)	91 (24.7)	0.144	178 (24.2)	91 (24.7)	0.901
Hypertension (%)	883 (60.4)	225 (61.1)	0.840	465 (63.2)	225 (61.1)	0.553
Diabetes (%)	286 (19.5)	63 (17.1)	0.324	120 (16.3)	63 (17.1)	0.797
Hypercholesterolemia (%)	980 (67.1)	234 (63.6)	0.228	476 (64.7)	234 (63.6)	0.773
Previous MI (%)	209 (14.3)	65 (17.7)	0.119	149 (20.2)	66 (17.9)	0.405
Previous PCI (%)	231 (15.8)	71 (19.4)	0.113	159 (21.6)	73 (19.8)	0.548
Previous CABG (%)	79 (5.4)	28 (7.6)	0.136	60 (8.2)	28 (7.6)	0.844
Previous stroke (%)	34 (2.3)	12 (3.3)	0.401	28 (3.8)	12 (3.3)	0.776
Previous CVD (%)	370 (25.3)	118 (32.1)	**0.010**	245 (33.3)	118 (32.1)	0.734
Valvular disease (%)	27 (1.8)	5 (1.4)	0.678	11 (1.5)	5 (1.4)	1.000
Chronic lung disease (%)	67 (4.6)	18 (4.9)	0.912	34 (4.6)	18 (4.9)	0.960
Family history of CAD (%)	397 (27.4)	97 (26.4)	0.759	173 (23.5)	97 (26.4)	0.334
Smoking (%)	562 (38.4)	125 (34.0)	0.132	243 (33.0)	125 (34.0)	0.804
BMI (kg/m^2^)	27.43 (4.44)	27.08 (4.73)	0.191	27.14 (4.33)	27.07 (4.72)	0.814
Haemoglobin (g/l), mean (SD)	137.11 (18.17)	136.28 (18.27)	0.445	136.56 (17.61)	136.24 (18.10)	0.777
Anemia (%)	305 (21.9)	82 (23.4)	0.577	159 (21.6)	88 (23.9)	0.429
Haematocrit, mean (SD)	40.36 (5.06)	40.28 (5.18)	0.787	40.26 (4.88)	40.26 (5.13)	0.990
eGFR, mean (SD)	92.75 (27.33)	89.18 (27.17)	**0.026**	89.65 (27.16)	89.22 (27.21)	0.804
LVEF, mean (SD)	55.41 (10.12)	55.92 (10.30)	0.407	55.66 (10.40)	55.88 (10.37)	0.733
ECG ischemia (%)	864 (62.0)	208 (58.3)	0.215	445 (60.5)	211 (57.3)	0.351
Heart rate (beats per minute), mean (SD)	75.33 (14.65)	76.40 (15.23)	0.215	75.94 (14.83)	76.40 (15.23)	0.634
Systolic blood pressure (mmHg), mean (SD)	132.78 (22.26)	133.02 (21.64)	0.851	133.08 (21.96)	132.88 (21.63)	0.885
GRACE score, mean (SD)	126.68 (27.85)	129.41 (29.15)	0.099	130.12 (27.84)	129.21 (29.07)	0.611
GRACE score >140 (%)	437 (30.8)	131 (36.0)	0.068	258 (36.4)	131 (36.0)	0.951
Killip class 2 (%)	90 (6.3)	26 (7.2)	0.626	45 (6.2)	26 (7.2)	0.658
Door-to-catheter time in minutes, median (IQR)	3.50 (1.80, 6.10)	16.80 (14.40, 19.50)	**<0.001**	3.50 (1.80, 6.00)	16.80 (14.40, 19.50)	**<0.001**

BMI = body mass index; CABG = coronary artery bypass graft; CAD = coronary artery disease; CVD = cardiovascular disease; eGFR = estimated glomerular filtration rate; GRACE = Global Registry of Acute Coronary Events; IQR = interquartile range; LVEF = left ventricular ejection fraction; MI = myocardial infarction; PCI = percutaneous intervention; SD = standard deviation.

**Table 2:** Treatment summary.

Treatment characteristics	Unmatched			Matched		
	<12 h (*n* = 1464)	12–24 h (*n* = 368)	*p*-value	<12 h (*n* = 736)	12–24 h (*n* = 368)	*p*-value
Angiography						
Revascularisation (%)			0.227			0.606
PCI—implantation of stent(s)	1246 (85.2)	302 (82.3)		624 (84.8)	302 (82.3)	
PCI—balloon dilatation only	69 (4.7)	20 (5.4)		37 (5.0)	20 (5.4)	
CABG	35 (2.4)	6 (1.6)		14 (1.9)	6 (1.6)	
No revascularisation	113 (7.7)	39 (10.6)		61 (8.3)	39 (10.6)	
AHA lesion class—worst (%)			0.781			0.742
1	344 (25.6)	88 (27.2)		166 (24.8)	88 (27.2)	
2	602 (44.9)	147 (45.4)		301 (44.9)	147 (45.4)	
3	203 (15.1)	42 (13.0)		101 (15.1)	42 (13.0)	
4	193 (14.4)	47 (14.5)		102 (15.2)	47 (14.5)	
Stent number (%)			0.112			0.214
0	0 (0.0)	1 (0.3)		0 (0.0)	1 (0.3)	
1	1012 (81.2)	238 (79.1)		508 (81.4)	238 (79.1)	
2	193 (15.5)	51 (16.9)		103 (16.5)	51 (16.9)	
3	32 (2.6)	7 (2.3)		10 (1.6)	7 (2.3)	
4	8 (0.6)	2 (0.7)		3 (0.5)	2 (0.7)	
5	1 (0.1)	2 (0.7)		0 (0.0)	2 (0.7)	
TIMI flow post-PCI (%)			0.808			0.829
0	11 (0.8)	2 (0.6)		6 (0.9)	2 (0.6)	
1	6 (0.5)	1 (0.3)		3 (0.5)	1 (0.3)	
2	20 (1.5)	7 (2.2)		10 (1.5)	7 (2.2)	
3	1283 (97.2)	310 (96.9)		643 (97.1)	310 (96.9)	
Thrombus (%)	305 (22.6)	43 (13.2)	**<0.001**	142 (21.1)	43 (13.2)	**0.003**
Aspiration (%)	193 (64.3)	25 (59.5)	0.663	87 (62.1)	25 (59.5)	0.900
Discharge medications						
Aspirin (%)	1444 (99.2)	362 (99.5)	0.841	725 (99.5)	362 (99.5)	1.000
Beta-blocker (%)	1175 (80.8)	295 (81.0)	0.979	600 (82.4)	295 (81.0)	0.636
ACE inhibitor (%)	1022 (70.3)	252 (69.2)	0.741	503 (69.1)	252 (69.2)	1.000
ARB (%)	260 (17.9)	70 (19.2)	0.602	143 (19.6)	70 (19.2)	0.935
Statin (%)	1445 (99.3)	358 (98.4)	0.149	723 (99.3)	358 (98.4)	0.239
P2Y_12_ agent (%)			**<0.001**			**0.011**
Clopidogrel	616 (44.4)	178 (52.2)		339 (48.4)	178 (52.2)	
Prasugrel	235 (17.0)	29 (8.5)		106 (15.1)	29 (8.5)	
Ticagrelor	535 (38.6)	134 (39.3)		255 (36.4)	134 (39.3)	
Admission timing						
Admission time (%)			**<0.001**			**<0.001**
Weekday (day)	936 (63.9)	112 (30.4)		488 (66.3)	112 (30.4)	
Weekday (night)	288 (19.7)	163 (44.3)		134 (18.2)	163 (44.3)	
Weekend	240 (16.4)	93 (25.3)		114 (15.5)	93 (25.3)	
Time of day, median (IQR)	10 : 19 (07 : 46–13 : 53)	18 : 02 (14 : 10–20 : 40)	**<0.001**	10 : 21 (07 : 55–13 : 52)	18 : 02 (14 : 10–20 : 40)	**<0.001**
Length of hospital stay in days, median (IQR)	2.12 (1.00, 4.51)	3.00 (1.16, 5.00)	**0.001**	2.00 (1.00, 4.39)	3.00 (1.16, 5.00)	**0.001**

ACE = angiotensin-converting enzyme; AHA = American Heart Association; ARB = angiotensin receptor blocker; CABG = coronary artery bypass graft; IQR = interquartile range; PCI = percutaneous intervention; SD = standard deviation; TIMI = thrombolysis in myocardial infarction.

## Data Availability

The clinical and procedural data used to support the findings of this study are included within the article and its supporting documents.

## Abbreviations

ACS: Acute coronary syndrome
CI: Confidence interval
DTC: Door-to-catheter
GRACE score: Global Registry of Acute Coronary Events
HR: Hazard ratio
LVEF: Left ventricular ejection fraction
MACE: Major adverse cardiovascular events
MI: Myocardial infarction
NSTE-ACS: Non-ST elevation acute coronary syndrome
NSTEMI: Non-ST elevation myocardial infarction
PCI: Percutaneous coronary intervention.

## Supplementary Materials

Supplementary Materials Supplementary Table 1: baseline characteristics with missing values: comparison of raw unmatched data with a single imputed data frame.
