# Supplementary material for: Optimal Timing of Invasive Coronary Angiography following NSTEMI
Source: J Interv Cardiol. 2020 Mar 3;2020:8513257. doi: 10.1155/2020/8513257 (PMC7073472; doi:10.1155/2020/8513257)
Supplement: Supplementary Materials — Supplementary Table 1: baseline characteristics with missing values: comparison of raw unmatched data with a single imputed data frame. [file 8513257.f1.docx]

**SUPPLEMENTARY MATERIAL**

**Supplementary Table 1. Baseline characteristics with missing values: comparison of raw unmatched data with a single imputed data frame.**

| BASELINE CHARACTERISTICS |  | RAW DATA | | | IMPUTED DATA | | |
| --- | --- | --- | --- | --- | --- | --- | --- |
|  | **Missing values (%)** | **<12h (n=1464)** | **12-24h (n=368)** | **p-value** | **<12h (n=1464)** | **12-24h (n=368)** | **p-value** |
| Diabetes (%) | 1 (0.1) | 286 (19.5) | 63 (17.1) | 0.32 | 286 (19.5) | 63 (17.1) | 0.33 |
| Hypertension (%) | 2 (0.1) | 883 (60.4) | 225 (61.1) | 0.84 | 885 (60.5) | 225 (61.1) | 0.86 |
| Hypercholesterolemia (%) | 3 (0.2) | 980 (67.1) | 234 (63.6) | 0.23 | 982 (67.1) | 234 (63.6) | 0.23 |
| Family history of CAD (%) | 16 (0.9) | 397 (27.4) | 97 (26.4) | 0.76 | 399 (27.3) | 97 (26.4) | 0.78 |
| Previous MI (%) | 3 (0.2) | 209 (14.3) | 65 (17.7) | 0.12 | 210 (14.3) | 66 (17.9) | 0.10 |
| Previous PCI (%) | 3 (0.2) | 231 (15.8) | 71 (19.4) | 0.11 | 231 (15.8) | 73 (19.8) | 0.07 |
| Previous CABG (%) | 1 (0.1) | 79 (5.4) | 28 (7.6) | 0.14 | 80 (5.5) | 28 (7.6) | 0.15 |
| Previous stroke (%) | 1 (0.1) | 34 (2.3) | 12 (3.3) | 0.40 | 34 (2.3) | 12 (3.3) | 0.40 |
| Previous CVD (%) | 1 (0.1) | 370 (25.3) | 118 (32.1) | 0.01 | 371 (25.3) | 118 (32.1) | 0.01 |
| Valvular disease (%) | 1 (0.1) | 27 (1.8) | 5 (1.4) | 0.68 | 27 (1.8) | 5 (1.4) | 0.68 |
| Chronic lung disease (%) | 3 (0.2) | 67 (4.6) | 18 (4.9) | 0.91 | 67 (4.6) | 18 (4.9) | 0.91 |
| Haemoglobin (g/l), mean (SD) | 87 (4.7) | 137.11 (18.17) | 136.28 (18.27) | 0.45 | 137.13 (18.16) | 136.24 (18.10) | 0.40 |
| Haematocrit, mean (SD) | 87 (4.7) | 40.36 (5.06) | 40.28 (5.18) | 0.79 | 40.37 (5.06) | 40.26 (5.13) | 0.72 |
| eGFR, mean (SD) | 25 (1.4) | 92.75 (27.33) | 89.18 (27.17) | **0.03** | 92.70 (27.42) | 89.22 (27.21) | **0.03** |
| ECG ischemia (%) | 82 (4.5) | 864 (62.0) | 208 (58.3) | 0.22 | 888 (60.7) | 211 (57.3) | 0.27 |
| BMI (kg/m^2^) | 17 (0.9) | 27.43 (4.44) | 27.08 (4.73) | 0.19 | 27.44 (4.48) | 27.07 (4.72) | 0.17 |
| GRACE score, mean (SD) | 50 (2.7) | 126.68 (27.85) | 129.41 (29.15) | 0.10 | 126.77 (27.78) | 129.21 (29.07) | 0.14 |
| LVEF, mean (SD) | 175 (9.6) | 55.41 (10.12) | 55.92 (10.30) | 0.41 | 55.22 (10.24) | 55.88 (10.37) | 0.27 |

Abbreviations: BMI = body mass index; CABG = coronary artery bypass graft; CAD = coronary artery disease; CVD = cardiovascular disease; eGFR = estimated glomerular filtration rate; GRACE = Global Registry of Acute Coronary Events; IQR = interquartile range; LVEF = left ventricular ejection fraction; MI = myocardial infarction; PCI = percutaneous intervention; SD = standard deviation;
